# Reassessing cumulative self-paced reading (SPR): Testing three variants shows cumulative SPR can be more useful than standard non-cumulative SPR for sentence-processing research, depending on presentation format

**DOI:** 10.3758/s13428-026-03044-7

**Published:** 2026-06-08

**Authors:** Hiroki Fujita

**Affiliations:** University of Potsdam, Potsdam, Germany

**Keywords:** Self-paced reading, Garden path, Number agreement, Sentence processing, Language comprehension

## Abstract

Self-paced reading (SPR) is widely used to investigate real-time sentence processing. In SPR, sentences can be presented either cumulatively, with previously presented words remaining visible, or non-cumulatively, with previous words disappearing. However, most prior research has avoided cumulative presentation, largely due to concerns that it allows readers to reveal multiple words through rapid key presses and then read them, thereby undermining the interpretability of reading times. As a result, cumulative SPR is widely assumed to be unsuitable for research on real-time sentence processing. The present study examines this assumption by comparing three cumulative SPR variants—ahead-visible cumulative SPR (AVC-SPR), non-ahead-visible cumulative SPR (NAVC-SPR), and partially cumulative SPR (PC-SPR)—with standard non-cumulative SPR (NC-SPR). In AVC-SPR, the positions of upcoming words are visually indicated; in NAVC-SPR, upcoming positions are not indicated; and in PC-SPR, upcoming positions are likewise not indicated, and accumulation is capped so that only a limited number of words remain visible. The four tasks were compared in terms of their sensitivity to detecting garden-path and number-mismatch effects. Clear effects were observed in all four tasks, with NAVC-SPR yielding the largest effect sizes. Power analyses further indicated that NAVC-SPR generally offers the highest prospective power to detect these effects. PC-SPR showed effect sizes similar to or larger than those in NC-SPR, and AVC-SPR was the least reliable task. Together, these findings challenge the assumption that cumulative presentation is unsuitable for studying real-time sentence processing and suggest that NAVC-SPR and PC-SPR are viable alternatives to NC-SPR. All cumulative SPR tasks, together with an R script for automated stimulus formatting, are openly available to facilitate their adoption.

## Introduction

The self-paced reading (SPR) task was developed in the late 1970 s (Aaronson & Scarborough, 1976; Mitchell & Green, 1978). Since then, it has become one of the most widely used chronometric methods for investigating the cognitive processes involved in real-time sentence comprehension (e.g., Boyce et al., 2020; Fujita, 2024b; Fujita & Yoshida, 2026; Gibson & Warren, 2004; Grodner et al., 2003; Kazanina et al., 2007; Nicenboim et al., 2018; Tabor & Hutchins, 2004; Wagers & Phillips, 2009; Witzel et al., 2012; Yoshida et al., 2013). In the moving-window SPR task (Just et al., 1982), sentences are presented in segments—typically word by word or phrase by phrase—on a computer screen. Participants advance through the sentence by pressing a key, usually the space bar. The time between key presses is recorded in milliseconds, and longer reading times are taken to reflect greater difficulty in processing the words (Just & Carpenter, 1980). Reading-time patterns are thus assumed to provide insight into how sentences are interpreted in real time.

SPR is relatively easy to implement and can be administered at low cost, factors that have contributed to its widespread use in sentence-processing research. Furthermore, SPR can be readily deployed online, enabling web-based data collection. This flexibility has further increased the use of SPR, particularly during and after the COVID-19 pandemic, when access to laboratory testing was restricted.

In SPR, sentences can be presented in two formats. In cumulative presentation, each sentence segment revealed by a key press remains visible until the end of the sentence. In non-cumulative presentation, each key press reveals the next segment while masking the previous one. Cumulative presentation may offer advantages over non-cumulative presentation because it allows readers to reread previous words, making the task more similar to natural reading conditions (Felser, 2021; Holmes et al., 1987; Kennedy & Murray, 1984). Allowing rereading may facilitate sentence comprehension, reduce variability in the data, and lower task demands, thereby reducing participants’ stress during the task. This may make SPR more suitable for populations such as second-language learners and young children, who may experience greater difficulty with non-cumulative SPR when earlier context is no longer visible. Furthermore, because readers may reread difficult material in natural reading, cumulative presentation may better approximate effect sizes obtained under conditions where rereading is possible or likely (e.g., Chen et al., 2025; Frazier & Rayner, 1982; Rayner et al., 2004; Sturt & Kwon, 2018; von der Malsburg & Vasishth, 2011).

Despite these potential advantages, prior work has predominantly used non-cumulative presentation, and cumulative presentation has rarely been adopted (Dussias et al., 2020; Jegerski, 2014). A primary concern about cumulative SPR is that it may encourage a rapid-unmasking strategy: participants may repeatedly press the key to reveal several upcoming words—or even the entire sentence—before beginning to read. If such a strategy is used, the intervals between key presses would be decoupled from the time required to process each segment, thereby undermining the interpretation of cumulative SPR latencies as measures of processing difficulty (Ferreira & Henderson, 1990; Just et al., 1982).

In sentence-processing research, the rapid-unmasking strategy was first noted by Just et al. (1982), who compared cumulative and non-cumulative SPR times with gaze-duration data reported by Just and Carpenter (1980). In these studies, participants read 15 texts (mean length = 132 words) drawn from Newsweek and Time. Just et al. reported largely similar word-level effects for gaze durations and non-cumulative SPR times (Table 1). For example, both gaze durations and non-cumulative SPR times increased for longer, less frequent words, for words central to a paragraph’s topic, and for sentence- or paragraph-final words. Cumulative SPR times showed broadly comparable patterns, but some effects (e.g., word frequency) were not clearly captured. Furthermore, correlations between gaze durations and non-cumulative SPR times were stronger than those between gaze durations and cumulative SPR times. Just et al. attributed this discrepancy to the use of a rapid-unmasking strategy under cumulative presentation.

**Table 1:** Sensitivity of reading times to lexical/discourse factors and correlations with gaze duration. Data were extracted from Just et al. (1982). For the factors other than “Mean reading time per word”, “Number of letters”, “Log frequency”, and “Correlation with gaze duration”, the values indicate additional reading times

	Methods (ms)		
Factors	Gaze duration	Non-cumulative SPR	Cumulative SPR
Mean reading time per word	239	441	451
Number of letters (per character)	32	15	10
Log frequency (per base-10 log unit)	33	15	–3
Beginning of line	16	53	50
Novel word (e.g., thermoluminescence)	692	1369	478
Digits	21	27	5
Last word in sentence	41	403	144
Last word in paragraph	154	719	635
First mention of topic	184	342	48
First content word	67	94	194
Correlation with mean gaze duration	1.00	.57	.40

Concerns about cumulative SPR remain widespread in sentence-processing research. A notable exception is Kennedy and Murray (1984), who suggest that cumulative presentation may be more effective than non-cumulative presentation for investigating real-time sentence processing. They compared the two presentation formats using garden-path and non-garden-path sentences (e.g., Frazier & Rayner, 1982; Pritchett, 1988, 1992). For example, they tested materials such as the following:

- *Non-garden-path*

While the teacher was reading the huge old book it fell off the table.

1. *Garden-path*

While the teacher was reading the huge old book fell off the table.

The term *garden path* or *garden-path effect* refers to the processing difficulty that arises from the misanalysis of locally ambiguous sentences. In (1a) and (1b), the noun phrase (NP) “*the huge old book*” is locally ambiguous: when encountered, it can be analyzed either as the direct object of “*reading*” (the object analysis) or as the subject of the matrix clause (the subject analysis). The object analysis is globally grammatical in (1a), but in (1b), it becomes unavailable at the disambiguating verb “*fell*”, where “*the huge old book*” is disambiguated as the subject of the matrix clause. Conversely, the subject analysis matches the globally grammatical structure of (1b) but conflicts with that of (1a), in which “*it*” serves as the subject of the matrix clause.

Substantial evidence suggests that, in sentences such as (1a) and (1b), the locally ambiguous noun phrase (NP) is initially analyzed as the direct object of the embedded verb (e.g., Clifton, 1993; Frazier & Rayner, 1982; Fujita, 2024b; Hopp, 2015; Jacob & Felser, 2016; Pickering & Traxler, 1998; Slattery et al., 2013; Sturt et al., 1999; Tabor et al., 2004). This parsing preference gives rise to processing difficulty—that is, a garden-path effect—when readers encounter the disambiguating region “*fell*” in (1b).

Using cumulative SPR, Kennedy and Murray (1984) observed a garden-path effect at the disambiguating region (“*fell off the*”), with mean reading times of 371 ms for (1a) and 476 ms for (1b). In contrast, their non-cumulative SPR condition showed little difference between the two sentence types ((1a): 303 ms; (1b): 315 ms), indicating no garden-path effect. The absence of a garden-path effect in their non-cumulative task makes these findings difficult to interpret, because many studies have reported robust garden-path effects with non-cumulative SPR (e.g., Fujita, 2024b; Grodner et al., 2003; Sturt et al., 1999). Nonetheless, their results raise the possibility that cumulative SPR can be sensitive to garden-path effects.

In summary, SPR is one of the most widely used methods for investigating real-time sentence processing. Although sentences can be presented either cumulatively or non-cumulatively, prior work has predominantly used non-cumulative presentation. Cumulative presentation has remained rare, primarily because of the concern that readers may adopt a rapid-unmasking strategy, rendering reading times unreliable as indicators of processing difficulty (Just et al., 1982). However, the evidence often cited in support of this concern does not in itself establish that cumulative presentation is unsuitable for sentence-processing research. Moreover, Kennedy and Murray (1984), despite the interpretive limitations of their overall findings, reported results consistent with the possibility that cumulative presentation can provide a sensitive measure of garden-path effects.

### The present study

**Fig. 1:**
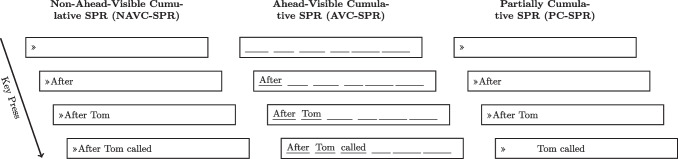
Three cumulative self-paced reading (SPR) variants. For illustration, the display limit in PC-SPR is set to two words

As discussed in the Introduction, non-cumulative presentation is far more common than cumulative presentation. Despite this strong preference, little direct comparative evidence is available regarding the performance of cumulative SPR relative to non-cumulative SPR. The present study is primarily methodological in nature, aiming to provide a direct comparison of cumulative and non-cumulative presentation and to assess the utility of cumulative presentation for sentence-processing research. Specifically, the study compares three cumulative SPR variants with standard non-cumulative presentation, using two well-established sentence-processing effects as diagnostic measures. The tasks are publicly available online at https://github.com/HirokiFujita1126/Cumulative-NonCumulativeSPRTask. The three cumulative SPR variants are illustrated in Fig. 1.

The first variant is non-ahead-visible cumulative SPR (NAVC-SPR). In this task, readers initially see a cursor (») indicating the position of the first word. Pressing the space bar reveals the first word, and subsequent key presses reveal the remaining words. Previously revealed words remain visible until the entire sentence has been read. Crucially, readers do not see any visual markers indicating the positions of upcoming words.

The second variant is ahead-visible cumulative SPR (AVC-SPR). In this task, each trial begins with a sequence of underlines marking the positions of all words in the sentence. As in NAVC-SPR, words are revealed sequentially with each key press, and previously revealed words remain visible until the end of the sentence.

The third variant is partially cumulative SPR (PC-SPR). This task functions like NAVC-SPR until the number of visible words reaches a preset cap, after which earlier words begin to disappear as new words are revealed. For example, in Fig. 1, NAVC-SPR and PC-SPR are identical up to the third display. However, in the fourth display, where the word “*called*” appears, the first word “*After*” disappears in PC-SPR because the display limit in this example is two words. Subsequent key presses reveal the remaining words while hiding earlier ones, thus maintaining the display limit.

I developed these three cumulative SPR tasks for the following reasons. First, Just et al. (1982) argued that their cumulative SPR yielded less reliable measures of processing times than their non-cumulative SPR because some participants rapidly revealed portions of sentences before reading them (i.e., they used a rapid-unmasking strategy). Just et al. did not specify the exact cumulative format they used; they described their procedure as follows: “successive words of the text were presented in their naturally occurring position from left to right on each line, with successive lines appearing below each other” (Just et al., 1982, p. 230). One possible reason their cumulative SPR yielded less reliable measures than their non-cumulative SPR is that it resembled AVC-SPR. This format may be particularly susceptible to rapid unmasking because readers can see both the number of words and the position of the final word before reading begins, potentially allowing them to estimate how many key presses are needed to reveal the entire sentence. AVC-SPR was included to test this hypothesis.

NAVC-SPR and PC-SPR were developed to test whether limiting advance information about sentence length and/or limiting how much preceding context remains visible can discourage rapid unmasking. NAVC-SPR does not display visual markers indicating upcoming word positions, so participants cannot know in advance how many words remain until the end of the sentence. This uncertainty may encourage more cautious key pressing, because pressing rapidly increases the risk of skipping past the end of the sentence and losing the opportunity to read it.

PC-SPR may further discourage rapid unmasking once the display cap is reached, while still allowing readers to reread a limited number of previous words. Importantly, by appropriately setting the display limit, PC-SPR can ensure that regions critical for sentence comprehension remain visible when subsequent words are encountered. For example, in the garden-path sentence in (1b) “*While the teacher was reading* *the huge old book fell off the table*”, a garden-path effect is expected at “*fell*” because the locally ambiguous NP “*the huge old book*” is initially misanalyzed as the direct object of “*reading*”. At “*fell*”, this NP is expected to be revised as the subject (Slattery et al., 2013), which requires dissociation from the embedded verb “*reading*” (Fodor & Inoue, 1998). Thus, during this revision, readers may attempt to reread the embedded verb and the locally ambiguous NP regions (e.g., Frazier & Rayner, 1982). If the goal is to measure a garden-path effect while allowing rereading of these regions and discouraging rapid unmasking, the display limit can be set to eight words. In that case, when readers reach “*fell*”, the embedded verb and the locally ambiguous noun remain visible for rereading, while earlier material has already begun to disappear (i.e., “*While the teacher*_*8*_* was*_*7*_* reading*_*6*_* the*_*5*_* huge*_*4*_* old*_*3*_* book*_*2*_* fell*_*1*_*…*”).

To evaluate the three cumulative SPR tasks relative to the non-cumulative SPR task, I examined whether they reliably capture two well-documented phenomena in sentence-processing research.

The first phenomenon is the garden-path effect, which arises in locally ambiguous sentences such as (1b). Garden-path effects have been extensively studied and widely observed (e.g., Crocker, 1996; Ferreira & Henderson, 1991; Fodor & Ferreira, 1998; Frazier, 1979; Fujita, 2021, 2023, 2024b, 2025b; Gibson, 1991; Jacob & Felser, 2016; Paape & Vasishth, 2022; Pritchett, 1992; Slattery et al., 2013; Sturt, 1997; Sturt et al., 1999; Sturt & Crocker, 1996; Tabor & Hutchins, 2004; Weinberg, 1999). Thus, if cumulative presentation is a viable method for sentence-processing research, it should be able to detect garden-path effects. Because sentences like (1b) may be particularly difficult to process (e.g., Fujita, 2024b; Sturt et al., 1999), rereading may play an important role, making cumulative presentation a particularly suitable method (Frazier & Rayner, 1982; Kennedy & Murray, 1984).

The second phenomenon is the number-mismatch effect, which arises when number agreement between a subject NP and a verb is violated. Consider, for example, (2).

(2) The sister of the girl recently were learning new skills online.

In English, a finite verb agrees in number with the corresponding subject NP (e.g., “*John was happy*”, not “*John were happy*”). In (2), this agreement is violated: the auxiliary verb “*were*” does not match the subject NP “*The sister...*” in number. Previous research provides substantial evidence that agreement violations lead to processing difficulty during real-time processing (e.g., Dillon et al., 2013; Fujita, 2024a, 2025a; Fujita & Cunnings, 2023, 2024; Fujita & Yoshida, 2024; Jäger et al., 2020; Parker & An, 2018; Pearlmutter et al., 1999; Sturt & Kwon, 2024; Wagers et al., 2009). Therefore, if cumulative presentation is a viable method for studying real-time sentence processing, it should reliably detect number-mismatch effects.

The utility of the three cumulative SPR tasks, relative to the non-cumulative SPR task, was evaluated using four criteria:

(i) Effect detection,
(ii) Effect size,
(iii) Effect localization, and
(iv) Prospective statistical power.

The first criterion concerns whether each task detects garden-path and number-mismatch effects. The second concerns the magnitude of these effects.

The third criterion concerns whether effects are localized to the critical region. In garden-path sentences, this is the disambiguating region (e.g., “*fell*” in (1b) “*While the teacher was reading the huge old book fell off the table*”); in subject–verb number-mismatch sentences, it is the verb region (e.g., “*were*” in (2) “*The sister of the girl recently were learning new skills online*”). In non-cumulative SPR, effects are often observed in a region following the critical region (e.g., “*learning*” in (2); Boyce et al., 2020; Fujita, 2025c; Witzel et al., 2012). If participants engage in rapid unmasking during cumulative SPR, effects may be further delayed. However, if NAVC-SPR and PC-SPR inhibit this strategy, they may yield effects that are at least as well localized as those in non-cumulative SPR.

The fourth criterion concerns reliability across different sample sizes—that is, how reliably cumulative SPR tasks detect garden-path and number-mismatch effects at different sample sizes relative to the non-cumulative SPR task. To address this criterion, I conducted a simulation-based power analysis and estimated prospective statistical power for detecting garden-path and number-mismatch effects across a range of sample sizes (20–100 participants), using the cumulative and non-cumulative SPR data collected in this study. The following section reports the results of the experiments and power analysis.

## Methods

I conducted four experiments to investigate whether garden-path and number-mismatch effects are detected in NAVC-SPR, AVC-SPR, PC-SPR, and standard non-cumulative SPR (NC-SPR). A sample pair of experimental sentences used to test garden-path effects is shown in (3a) and (3b).

- *Unambiguous*

After the man woke up, the woman in the bedroom started to watch the news.

(3b) *Ambiguous*

After the man woke up the woman in the bedroom started to watch the news.

In (3b), the NP “*the woman in the bedroom*” can be locally analyzed as the direct object of “*woke up*”. However, at the disambiguating region “*started*”, this object analysis becomes unavailable because the NP is disambiguated as the subject of the matrix clause. Sentence (3a) does not locally permit the object analysis because the comma following “*woke up*” rules it out. Previous research suggests that readers initially prefer the object analysis in (3b), leading to a garden-path effect at or immediately following the disambiguating region “*started*” (e.g., Frazier & Rayner, 1982). Observing this garden-path effect in cumulative SPR would indicate that cumulative presentation is sensitive to online sentence-processing difficulty.

I also examined a potential advantage of cumulative over non-cumulative SPR: allowing rereading may aid comprehension, particularly for sentences that are difficult to process. To examine this possibility, I included comprehension questions targeting the initial misinterpretation of locally ambiguous sentences, such as “*Did the man wake up the woman?*” for (3a) and (3b). The correct answer is “*no*” because the sentences mean that the man woke up, not that he woke up the woman. However, previous research suggests that participants often answer “*yes*” to such questions in ambiguous sentences, indicating that initial misinterpretations can persist after disambiguation (*lingering misinterpretation*; see Christianson et al., 2001; Fujita & Cunnings, 2020, 2021a, b; Slattery et al., 2013; Sturt, 2007). If rereading facilitates sentence comprehension, accuracy should be higher in cumulative than in non-cumulative SPR, particularly for ambiguous sentences.

A sample pair of experimental sentences used to test number-mismatch effects is shown in (4a) and (4b).

- *Number Match*

John and Mary said that the sisters of the girl recently were learning new skills online.

(4b) *Number Mismatch*

John and Mary said that the sister of the girl recently were learning new skills online.

In (4a) and (4b), the subject NP “*the sister(s)…*” and the auxiliary verb “*were*” form a subject–verb dependency. In (4a), they match in number, whereas in (4b), they do not. Previous research suggests that number mismatch causes processing difficulty during real-time processing (e.g., Wagers et al., 2009). Thus, if cumulative presentation is a viable method for investigating real-time sentence processing, it should reliably detect number-mismatch effects.

### Participants

For each of the four tasks (NAVC-SPR, AVC-SPR, PC-SPR, and NC-SPR), 100 participants were recruited (400 in total) via Prolific (https://www.prolific.com/). All participants were born and raised in the UK, spoke English as their first language, and were British citizens.

### Materials

Each task included 14 sets of unambiguous/ambiguous sentences like (3a) and (3b), and 14 sets of number-match/mismatch sentences like (4a) and (4b). Each task also included 60 filler sentences. None of the filler sentences contained local ambiguities of the type shown in (3b) or number mismatches of the type shown in (4b). Each experimental and filler sentence was followed by a yes/no comprehension question. For unambiguous/ambiguous items, the questions targeted lingering misinterpretations. For all other sentences, the questions probed different parts of the sentences.

### Procedures

The cumulative SPR tasks followed the procedures shown in Fig. 1. The NC-SPR task used a standard moving-window procedure in which each key press revealed the next word while masking the previous one. For PC-SPR, the display limit was set to nine words. This limit allowed participants to reread the locally ambiguous NP (“*the woman…*”), the embedded verb (“*woke up*”), and the embedded subject noun (“*man*”) when they reached the disambiguating region in unambiguous/ambiguous sentences (“*After the man*_*9*_* woke*_*8*_* up*_*7*_*(,) the*_*6*_* woman*_*5*_* in*_*4*_* the*_*3*_* bedroom*_*2*_* started*_*1*_*…*”). In number-match/mismatch sentences, it allowed rereading of the subject NP in the embedded clause (e.g., “*the sister…*”) when participants reached the verb region (“*John and Mary said*_*9*_* that*_*8*_* the*_*7*_* sister(s)*_*6*_* of*_*5*_* the*_*4*_* girl*_*3*_* recently*_*2*_* were*_*1*_*…*”). Experimental sentences were distributed using a Latin square design and presented in a pseudo-random order. Each task began with practice trials.

To create the tasks, I developed an automation script, which is openly available at https://github.com/HirokiFujita1126/ibfp. The script formats experimental stimuli for use with Ibex (https://github.com/addrummond/ibex/tree/master). The tasks were administered using PCIbex Farm (Zehr & Schwarz, 2018).

### Data analysis

Log-transformed reading times were analyzed using linear mixed-effects models fitted with lme4 (Bates et al., 2015) in R (R Core Team, 2025). For unambiguous/ambiguous sentences (3a/b), reading times at the disambiguating region (“*started*”) and the post-disambiguating region (“*to*”) were analyzed separately. For number-match/mismatch sentences (4a/b), reading times at the verb region (“*were*”) and the post-verb region (“*learning*”) were likewise analyzed separately.

I analyzed two datasets:

(i) One in which raw reading times shorter than 200 ms or longer than 10,000 ms were excluded, and
(ii) One in which no reading times were removed.^1^

These analyses yielded similar results. Following a reviewer’s recommendation, I report results for the dataset with no reading-time exclusions below.

Garden-path and number-mismatch effects were analyzed in separate mixed-effects models. For each phenomenon, data from all four tasks were analyzed within a single model by including Task as a predictor. Task was coded using treatment coding with NC‑SPR as the reference level, yielding three dummy predictors that compare each cumulative SPR task to NC‑SP

**Table:** 

	Comparison		
Level	NAVC vs. NC	AVC vs. NC	PC vs. NC
NC-SPR	0	0	0
NAVC-SPR	1	0	0
AVC-SPR	0	1	0
PC-SPR	0	0	1

With this treatment coding, the main effects of Task indicate differences in mean reading time between each cumulative SPR task and NC‑SPR at the midpoint of the sum-coded condition predictor—that is, averaged across the Ambiguity or Number conditions (see below).

For unambiguous/ambiguous sentences, Ambiguity (unambiguous vs. ambiguous) was included as a predictor; for number-match/mismatch sentences, Number (match vs. mismatch) was included. Both predictors were sum-coded (−1/+1). With this coding, the main effect of Ambiguity (or Number) represents the garden-path (or number-mismatch) effect for NC‑SPR, the reference level of Task. In the Results section, I report simple Ambiguity (or Number) effects for all four tasks.

Each model also included the relevant interaction (Ambiguity × Task or Number × Task). This interaction tests whether the garden-path or number-mismatch effect differs between each cumulative task and NC‑SPR, with its estimates quantifying the corresponding differences in effect size relative to NC‑SPR.

Each model was initially fitted with a full random-effects structure: varying intercepts for participants and items, varying slopes for Ambiguity or Number by participants and items, and correlation parameters among these participant- and item-specific coefficients. When the initial model failed to converge, the correlation parameters were first set to zero. If the model still did not converge, it was simplified by iteratively removing the participant- or item-specific slope term with the smallest estimated variance component and refitting after each step until convergence was achieved.

Comprehension accuracy for unambiguous/ambiguous sentences was analyzed in a similar way, but using generalized linear mixed-effects models with a logistic link function.

## Results

Mean comprehension accuracy for number-match/mismatch and filler sentences was high across tasks:

- 91% (range 72–99%) for NAVC-SPR
- 92% (range 72–100%) for AVC-SPR
- 91% (range 80–99%) for PC-SPR
- 90% (range 70–100%) for NC-SPR

These high accuracy rates indicate that participants were generally attentive during the tasks.

### Unambiguous/Ambiguous sentences: Reading times

**Fig. 2:**
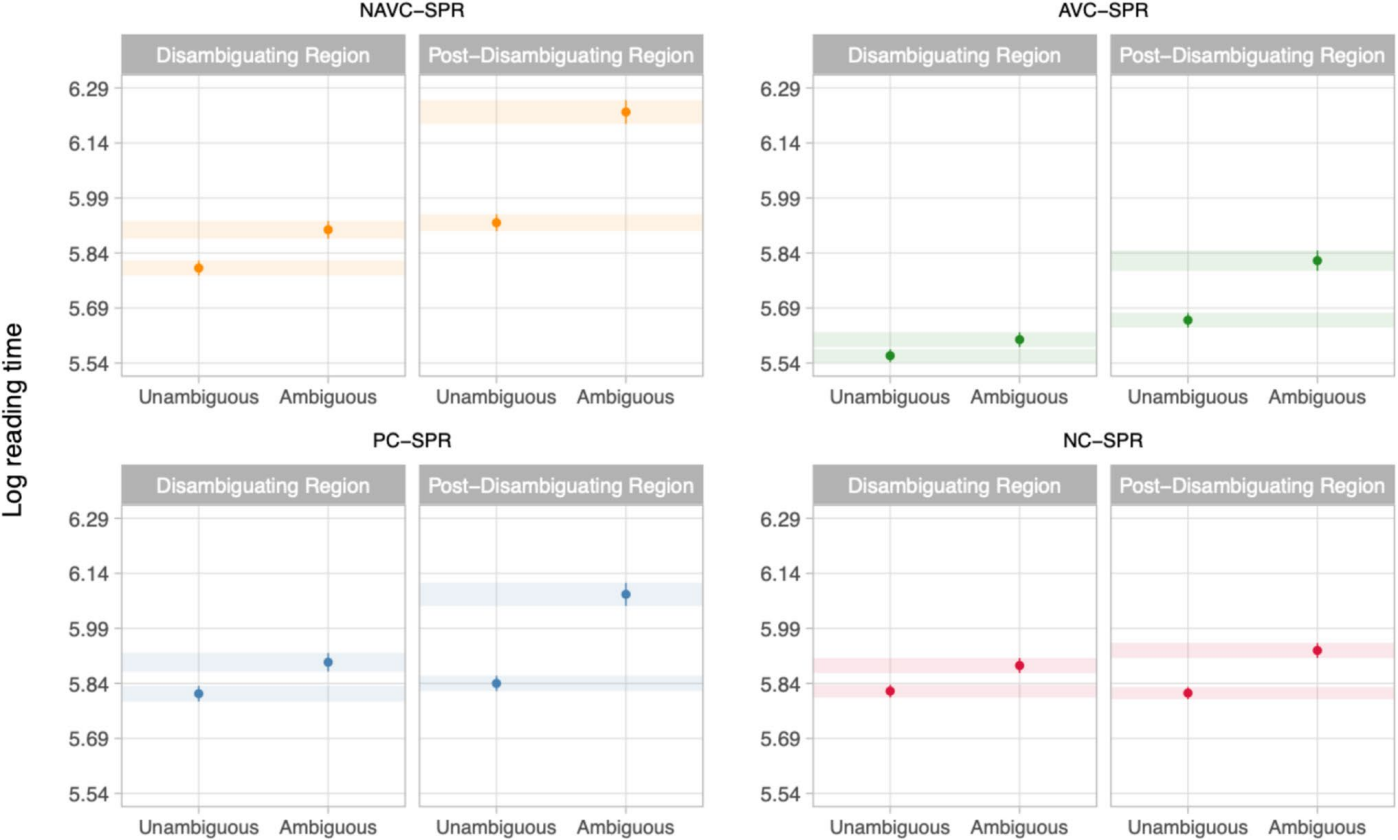
Log-transformed reading times at the disambiguating and post-disambiguating regions in unambiguous and ambiguous sentences. *Note*. *Error bars* *are standard errors. NAVC-SPR = non-ahead-visible cumulative SPR; AVC-SPR = ahead-visible cumulative SPR; PC-SPR = partially cumulative SPR; NC-SPR = non-cumulative SPR*

**Fig. 3:**
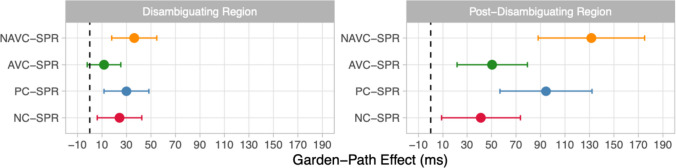
Estimated garden-path effects with 95% compatibility intervals. *Note. Positive values on the x-axis indicate longer reading times for ambiguous than for unambiguous sentences (i.e., garden-path effects). NAVC-SPR = non-ahead-visible cumulative SPR; AVC-SPR = ahead-visible cumulative SPR; PC-SPR = partially cumulative SPR; NC-SPR = non-cumulative SPR*

Figure 2 shows log-transformed reading times at the disambiguating and post-disambiguating regions. Figure 3 shows estimated garden-path effects with 95% compatibility intervals (CIs). Inferential statistics are summarized in Table 2, with key effects highlighted in the text. For interpretability, I report (i) ΔRT, the back-transformed model-predicted difference in reading time between conditions (e.g., ${RT}_{ambiguous}-{RT}_{unambiguous}$, i.e., a garden-path effect), together with 95% CIs; and (ii) the corresponding multiplicative effect as a ratio, with *t*-statistics on the log scale. For interaction effects, I report ΔΔRT (i.e., the difference between ΔRTs across Task) and the corresponding ratio-of-ratios.

**Table 2:** Inferential statistics for unambiguous and ambiguous sentences (reading time). *Note. Estimates (Ests) and 95% compatibility intervals (CIs) are back-transformed. t statistics are reported on the log scale. NAVC = non-ahead-visible cumulative SPR; AVC = ahead-visible cumulative SPR; PC = partially cumulative SPR; NC = non-cumulative SPR; GP = garden-path effect*

	Disambiguating region				Post-disambiguating region			
Predictor	Est	95% CI	Ratio	*t*	Est	95% CI	Ratio	*t*
GP_NAVC_	36	[18, 55]	1.11	4.05	132	[88, 175]	1.35	6.93
GP_AVC_	12	[–2, 25]	1.04	1.70	50	[22, 79]	1.18	3.72
GP_PC_	30	[12, 48]	1.09	3.32	94	[57, 132]	1.27	5.57
GP_NC_	24	[6, 42]	1.07	2.70	41	[9, 74]	1.12	2.66
Task_NAVC vs. NC_	–1	[–33, 31]	1.00	–0.06	79	[39, 120]	1.22	4.04
Task_AVC vs. NC_	–83	[–112, –54]	0.76	–5.86	–44	[–78, –11]	0.87	–2.67
Task_PC vs. NC_	0	[–32, 32]	1.00	0.02	33	[–4, 71]	1.09	1.79
GP × Task_NAVC vs. NC_	12	[–14, 38]	1.04	0.96	90	[47, 134]	1.20	3.88
GP × Task_AVC vs. NC_	–13	[–35, 10]	0.97	–0.71	9	[–25, 44]	1.05	0.96
GP × Task_PC vs. NC_	6	[–20, 31]	1.02	0.44	53	[14, 93]	1.14	2.65

#### Disambiguating region

Garden-path effects were observed for NAVC-SPR (ΔRT = 36 ms, 95% CI [18, 55] ms; ratio = 1.11; log-scale *t* = 4.05), PC-SPR (ΔRT = 30 ms, 95% CI [12, 48] ms; ratio = 1.09; log-scale *t* = 3.32), and NC-SPR (ΔRT = 24 ms, 95% CI [6, 42] ms; ratio = 1.07; log-scale *t* = 2.70). Evidence for a garden-path effect was weak for AVC-SPR (ΔRT = 12 ms, 95% CI [–2, 25] ms; ratio = 1.04; log-scale *t* = 1.70), although there was no clear interaction between Ambiguity and Task for the AVC-SPR vs. NC-SPR comparison (ΔΔRT = –13 ms, 95% CI [–35, 10] ms; ratio-of-ratios = 0.97; log-scale *t* = –0.71).

There was also a main effect of Task for the AVC-SPR vs. NC-SPR comparison (ΔRT = –83 ms, 95% CI [–112, –54] ms; ratio = 0.76; log-scale *t* = –5.86), indicating shorter reading times in AVC-SPR than in NC-SPR. The other comparisons—NAVC-SPR vs. NC-SPR and PC-SPR vs. NC-SPR—did not show clear effects (both |*t*| < 0.06).

#### Post-disambiguating region

Garden-path effects were observed for all tasks: NAVC-SPR (ΔRT = 132 ms, 95% CI [88, 175] ms; ratio = 1.35; log-scale *t* = 6.93), AVC-SPR (ΔRT = 50 ms, 95% CI [22, 79] ms; ratio = 1.18; log-scale *t* = 3.72), PC-SPR (ΔRT = 94 ms, 95% CI [57, 132] ms; ratio = 1.27; log-scale *t* = 5.57), and NC-SPR (ΔRT = 41 ms, 95% CI [9, 74] ms; ratio = 1.12; log-scale *t* = 2.66).

There were also interactions between Ambiguity and Task for the NAVC-SPR vs. NC-SPR comparison (ΔΔRT = 90 ms, 95% CI [47, 134] ms; ratio-of-ratios = 1.20; log-scale *t* = 3.88) and for the PC-SPR vs. NC-SPR comparison (ΔΔRT = 53 ms, 95% CI [14, 93] ms; ratio-of-ratios = 1.14; log-scale *t* = 2.65). These interactions indicate that garden-path effects were larger in NAVC-SPR and PC-SPR than in NC-SPR.

Main effects of Task were also observed for the NAVC-SPR vs. NC-SPR comparison (ΔRT = 79 ms, 95% CI [39, 120] ms; ratio = 1.22; log-scale *t* = 4.04) and for the AVC-SPR vs. NC-SPR comparison (ΔRT = –44 ms, 95% CI [–78, –11] ms; ratio = 0.87; log-scale *t* = –2.67), indicating longer overall reading times in NAVC-SPR and shorter overall reading times in AVC-SPR than in NC-SPR.

Summary (reading times for unambiguous and ambiguous sentences):

(i) Garden-path effects were observed in all tasks, but evidence was weak at the disambiguating region in AVC-SPR.
(ii) Garden-path effects were larger in NAVC-SPR and PC-SPR than in NC-SPR.
(iii) Overall reading times were shorter in AVC-SPR and longer in NAVC-SPR than in NC-SPR.

### Unambiguous/Ambiguous sentences: comprehension accuracy

**Fig. 4:**
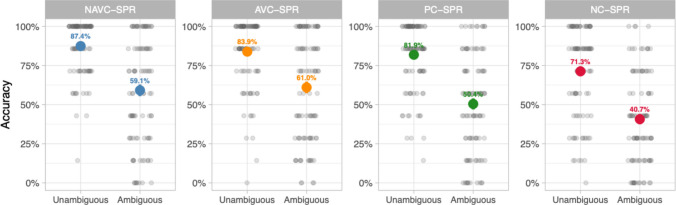
Raw comprehension accuracy for unambiguous and ambiguous sentences*. Note. Small grey dots indicate individual participant means. NAVC-SPR = non-ahead-visible cumulative SPR; AVC-SPR = ahead-visible cumulative SPR; PC-SPR = partially cumulative SPR; NC-SPR = non-cumulative SPR*

Figure 4 shows mean comprehension accuracy for unambiguous and ambiguous sentences. Below, I report (i) Δp, the back-transformed model-predicted difference in probability (in percentage points) between conditions (e.g., ${p}_{ambiguous}-{p}_{unambiguous}$), together with 95% CIs; and (ii) the corresponding multiplicative effect as an odds ratio (OR), with *z*-statistics on the logit scale. Correct responses are coded as 1 and incorrect responses as 0.

Ambiguity effects were observed for all tasks: NAVC-SPR (Δp = –29 pp, 95% CI [–43, –14] pp; OR = 0.11; logit-scale *z* = –8.37), AVC-SPR (Δp = –24 pp, 95% CI [–37, –11] pp; OR = 0.18; logit-scale *z* = –6.69), PC-SPR (Δp = –40 pp, 95% CI [–55, –25] pp; OR = 0.11; logit-scale *z* = –8.83), and NC-SPR (Δp = –45 pp, 95% CI [–56, –34] pp; OR = 0.14; logit-scale *z* = –8.31). These effects indicate lower comprehension accuracy in the ambiguous condition than in the unambiguous condition (i.e., lingering misinterpretation).

Main effects of Task were also observed for all comparisons: NAVC-SPR vs. NC-SPR (Δp = 27 pp, 95% CI [15, 38] pp; OR = 4.11; logit-scale *z* = 5.68), AVC-SPR vs. NC-SPR (Δp = 25 pp, 95% CI [14, 36] pp; OR = 3.67; logit-scale *z* = 5.26), and PC-SPR vs. NC-SPR (Δp = 17 pp, 95% CI [6, 27] pp; OR = 2.14; logit-scale *z* = 3.12), indicating higher accuracy in cumulative SPR (NAVC-SPR, AVC-SPR, PC-SPR) than in non-cumulative SPR (NC-SPR).

There was no clear evidence for Ambiguity × Task interactions (all |*z*| < 0.97).

Summary (comprehension accuracy for unambiguous and ambiguous sentences):

(i) Accuracy was lower for ambiguous sentences than for unambiguous sentences in all tasks (i.e., lingering misinterpretation).
(ii) Accuracy was higher in cumulative SPR (NAVC-SPR, AVC-SPR, PC-SPR) than in non-cumulative SPR (NC-SPR).

### Number-match/mismatch sentences: reading times

**Fig. 5:**
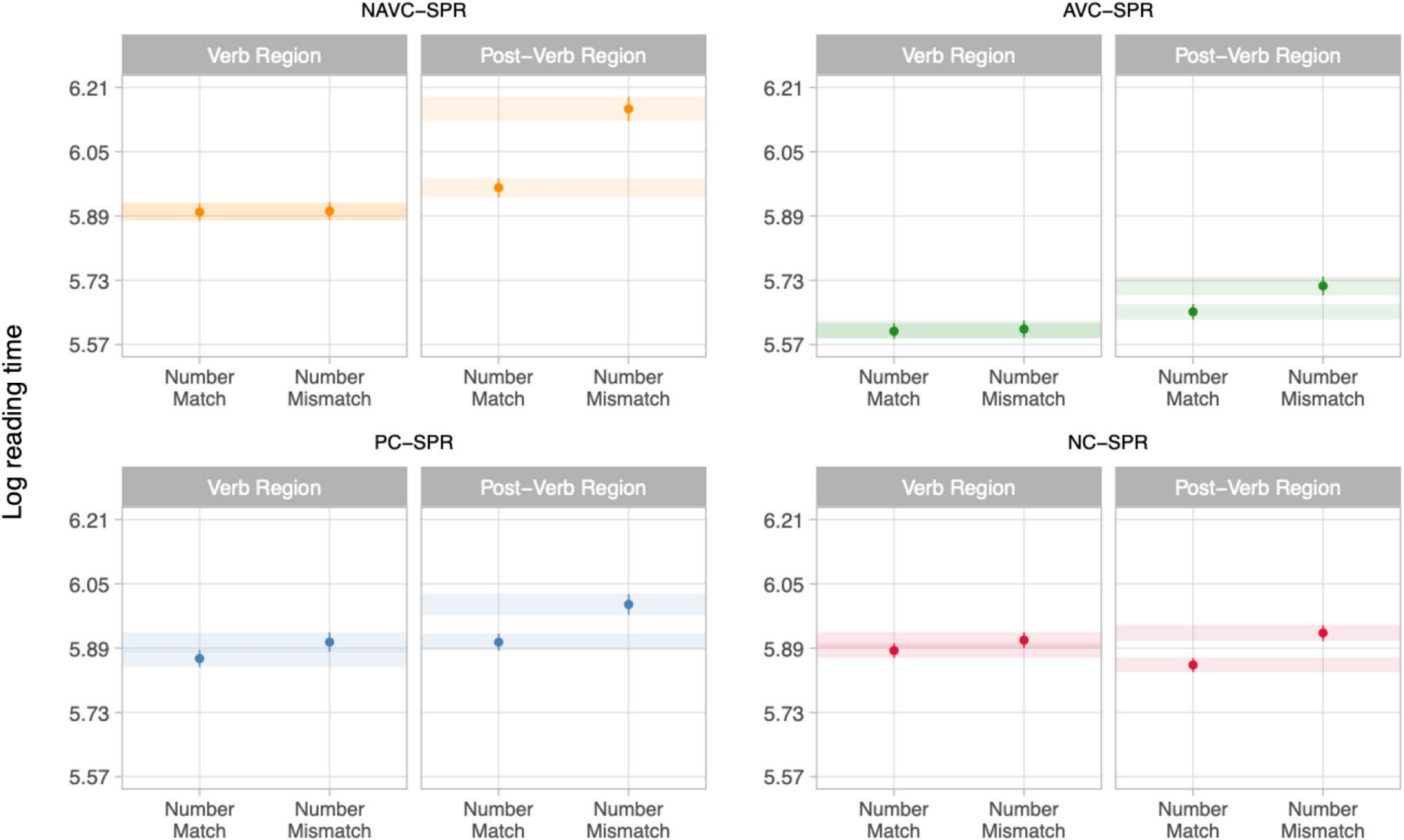
Log-transformed reading times at the verb and post-verb regions in number-match and number-mismatch sentences. *Note.* *Error bars are standard errors. NAVC-SPR = non-ahead-visible cumulative SPR; AVC-SPR = ahead-visible cumulative SPR; PC-SPR = partially cumulative SPR; NC-SPR = non-cumulative SPR*

**Fig. 6:**
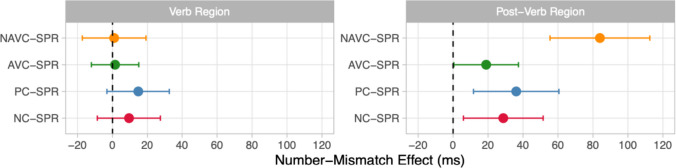
Estimated number-mismatch effects with 95% compatibility intervals. *Note. Positive values on the x-axis indicate longer reading times for number-mismatch than for number-match sentences (i.e., number-mismatch effects). NAVC-SPR = non-ahead-visible cumulative SPR; AVC-SPR = ahead-visible cumulative SPR; PC-SPR = partially cumulative SPR; NC-SPR = non-cumulative SPR*

Log-transformed reading times at the verb and post-verb regions are illustrated in Figure 5. Figure 6 shows estimated number-mismatch effects, and inferential statistics are summarized in Table 3. Below, I report back-transformed model-predicted differences in reading time between conditions (e.g., ${RT}_{mismatch}-{RT}_{match}$, i.e., a number-mismatch effect), together with 95% CIs, and multiplicative effects as ratios, with *t*-statistics on the log scale.

**Table 3:** Inferential statistics for number-match and number-mismatch sentences (reading time). *Note. Estimates (Ests) and 95% compatibility intervals (CIs) are back-transformed. t statistics are reported on the log scale. NAVC = non-ahead-visible cumulative SPR; AVC = ahead-visible cumulative SPR; PC = partially cumulative SPR; NC = non-cumulative SPR; NM = number-mismatch effect*

	Verb Region				Post-Verb Region			
Predictor	Est	95% CI	Ratio	*t*	Est	95% CI	Ratio	*t*
NM_NAVC_	1	[–17, 19]	1.00	0.10	84	[55, 113]	1.22	6.30
NM_AVC_	1	[–12, 15]	1.01	0.21	19	[0.4, 37]	1.07	2.07
NM_PC_	15	[–3, 33]	1.04	1.62	36	[12, 61]	1.10	3.02
NM_NC_	9	[–9, 28]	1.03	1.03	29	[6, 52]	1.08	2.56
Task_NAVC vs. NC_	1	[–32, 35]	1.00	0.09	67	[30, 104]	1.19	3.59
Task_AVC vs. NC_	–92	[–121, –63]	0.75	–6.30	–67	[–98, –36]	0.82	–4.29
Task_PC vs. NC_	–4	[–37, 28]	0.99	–0.27	24	[–11, 59]	1.07	1.34
NM × Task_NAVC vs. NC_	–9	[–34, 17]	0.98	–0.66	55	[19, 91]	1.12	2.68
NM × Task_AVC vs. NC_	–8	[–31, 15]	0.98	–0.58	–10	[–39, 19]	0.98	–0.35
NM × Task_PC vs. NC_	5	[–20, 31]	1.02	0.42	7	[–26, 40]	1.01	0.33

#### Verb region

Clear number-mismatch effects were not observed in any task: NAVC-SPR (ΔRT = 1 ms, 95% CI [–17, 19] ms; ratio = 1.00; log-scale *t* = 0.10), AVC-SPR (ΔRT = 1 ms, 95% CI [–12, 15] ms; ratio = 1.01; log-scale *t* = 0.21), PC-SPR (ΔRT = 15 ms, 95% CI [–3, 33] ms; ratio = 1.04; log-scale *t* = 1.62), and NC-SPR (ΔRT = 9 ms, 95% CI [–9, 28] ms; ratio = 1.03; log-scale *t* = 1.03).

There was also no clear evidence of Number × Task interactions (all |*t*| < 0.66).

A main effect of Task was observed for the AVC-SPR vs. NC-SPR comparison (ΔRT = –92 ms, 95% CI [–121, –63] ms; ratio = 0.75; log-scale *t* = –6.30), indicating shorter reading times in AVC-SPR than in NC-SPR.

#### Post-verb region

Number-mismatch effects were observed for all tasks: NAVC-SPR (ΔRT = 84 ms, 95% CI [55, 113] ms; ratio = 1.22; log-scale *t* = 6.30), AVC-SPR (ΔRT = 19 ms, 95% CI [0.4, 37] ms; ratio = 1.07; log-scale *t* = 2.07), PC-SPR (ΔRT = 36 ms, 95% CI [12, 61] ms; ratio = 1.10; log-scale *t* = 3.02), and NC-SPR (ΔRT = 29 ms, 95% CI [6, 52] ms; ratio = 1.08; log-scale *t* = 2.56).

There was also an interaction between Number and Task for the NAVC-SPR vs. NC-SPR comparison (ΔΔRT = 55 ms, 95% CI [19, 91] ms; ratio-of-ratios = 1.12; log-scale *t* = 2.68), indicating a larger number-mismatch effect in NAVC-SPR than in NC-SPR.

Main effects of Task were observed for the NAVC-SPR vs. NC-SPR comparison (ΔRT = 67 ms, 95% CI [30, 104] ms; ratio = 1.19; log-scale *t* = 3.59) and for the AVC-SPR vs. NC-SPR comparison (ΔRT = –67 ms, 95% CI [–98, –36] ms; ratio = 0.82; log-scale *t* = –4.29). These effects indicate longer reading times in NAVC-SPR than in NC-SPR and shorter reading times in AVC-SPR than in NC-SPR.

Summary (reading times for number-match and number-mismatch sentences):

(i) Number-mismatch effects were observed in all tasks.
(ii) Number-mismatch effects were larger in NAVC-SPR than in NC-SPR.
(iii) Overall reading times were shorter in AVC-SPR and longer in NAVC-SPR than in NC-SPR.

These results are compatible with those for unambiguous and ambiguous sentences.

### Power analysis

Prospective power was estimated using the following simulation procedure (Vasishth et al., 2022; Vasishth & Gelman, 2021). First, Bayesian multilevel regression models with a lognormal likelihood were fitted to reading times using brms (Bürkner, 2017) in R. Models were fitted separately for each region (disambiguating region, post-disambiguating region, verb region, and post-verb regions), for each phenomenon (garden-path effect and number-mismatch effect), and for each task (NAVC-SPR, AVC-SPR, PC-SPR, and NC-SPR). Each model included the relevant predictor (Ambiguity or Number) and by-participant and by-item varying intercepts and slopes with full variance-covariance matrices.

Priors were specified as follows:

- Intercept: Normal (6, 2)
- Slope: Normal (0, 3)
- Sigma: Normal (0, 1)
- SD: Normal (0, 1)
- Correlation: LKJ (2) (Lewandowski et al., 2009)

Each model was run with four chains of 4000 iterations, with the first 2000 iterations discarded as warm-up. Convergence was assessed using R-hat and visual inspection of trace plots; all models used for the power analysis converged satisfactorily.

Next, posterior samples of the fixed effects and variance components were used to generate datasets. For each sample size (20, 40, 60, 80, or 100 participants), each region, and each task, I generated 1000 datasets using 14 item sets (matching the experiments). For each simulated dataset, I then fitted a linear mixed-effects model using lme4 to the log-transformed reading time. In total, 80,000 models were fitted (1000 datasets × 5 sample sizes × 4 regions × 4 tasks).

**Fig. 7:**
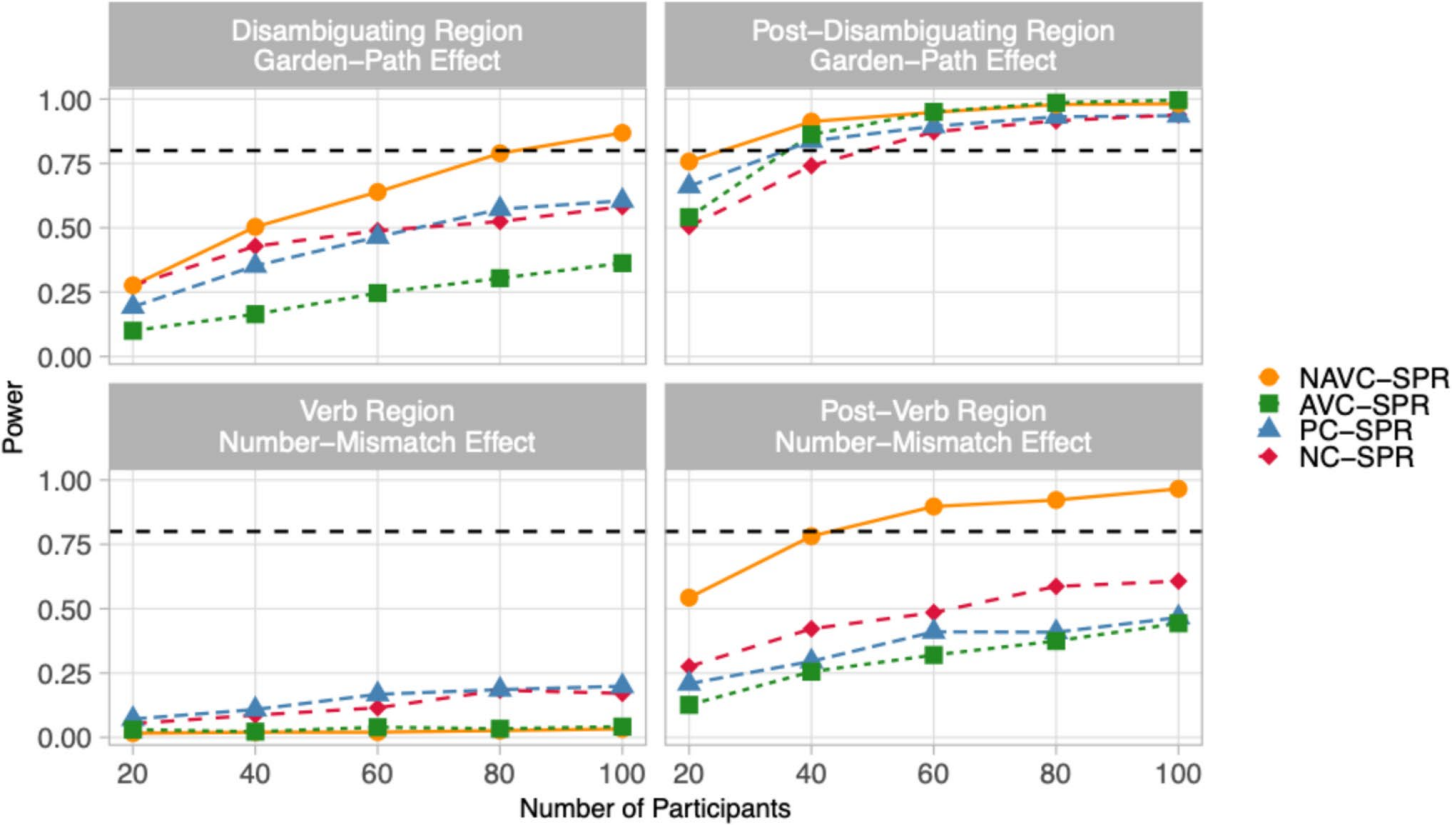
Prospective power for detecting garden-path and number-mismatch effects across sample sizes. *Note. The number of item sets was fixed at 14. The black dotted line marks power = 0.80. NAVC-SPR = non-ahead-visible cumulative SPR; AVC-SPR = ahead-visible cumulative SPR; PC-SPR = partially cumulative SPR; NC-SPR = non-cumulative SPR*

Prospective power was defined as the proportion of fitted models in which the *t*-value for the critical predictor (Ambiguity or Number) was ≥ 2.00 in the predicted direction (i.e., longer reading times for ambiguous sentences than for unambiguous sentences, or for number-mismatch sentences than for number-match sentences). Models that failed to converge were excluded from the power calculation (< 1%). Results are shown in Fig. 7.

#### Garden-path effect

The power analysis indicated that NAVC‑SPR had the highest estimated power to detect garden‑path effects. At the disambiguating region, NAVC-SPR yielded the highest estimated power across sample sizes (20–100 participants), exceeding NC‑SPR throughout. For smaller samples 20–60 participants, NC‑SPR had the second-highest power; at larger samples (80–100 participants), PC‑SPR exceeded NC‑SPR. AVC‑SPR showed the lowest power.

At the post‑disambiguating region, all cumulative SPR tasks (NAVC‑SPR, PC‑SPR, AVC‑SPR) generally had higher power than NC‑SPR.

#### Number-mismatch effect

At the verb region, estimated power was generally low across tasks; PC‑SPR showed the highest power, consistent with its number‑mismatch effect being more localized to the verb region.

At the post‑verb region, NAVC‑SPR consistently yielded the highest power, followed by NC‑SPR, PC‑SPR, and AVC‑SPR.

## General discussion

I conducted four experiments to investigate the utility of cumulative SPR for sentence-processing research, relative to non-cumulative SPR.

In previous SPR research, non-cumulative presentation has been predominantly used, whereas cumulative presentation has been used only rarely. The primary reason is that, when a sentence is displayed cumulatively, readers may employ a rapid-unmasking strategy—revealing several upcoming words, or a substantial portion of the sentence, before beginning to read. As a result, the reading time recorded at each region may not accurately reflect the actual processing time for that region (Ferreira & Henderson, 1990; Just et al., 1982). The present study examined whether this concern is justified and whether NAVC-SPR and PC-SPR discourage the rapid-unmasking strategy. I first discuss the utility of cumulative SPR relative to non-cumulative SPR and then revisit the findings of Just et al. (1982).

### Is cumulative SPR unsuitable for investigating real-time sentence processing?

**Fig. 8:**
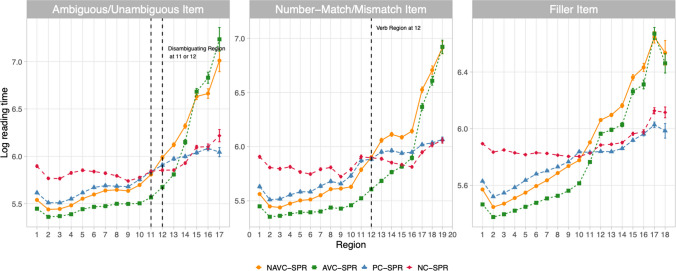
Log-transformed reading times across regions for experimental and filler sentences. *Note. Error bars are standard errors*

The present results suggest that some readers may engage in a rapid-unmasking strategy in AVC-SPR, where upcoming word positions are indicated. This inference is primarily based on the observation that garden-path effects at the disambiguating region were less clearly detected in AVC-SPR than in the other tasks and that AVC-SPR had the lowest estimated power to detect number-mismatch effects. Furthermore, reading times in AVC-SPR were generally shorter than in the other tasks at both the critical and spillover regions. This pattern was also evident at earlier regions. Figure 8 illustrates reading times across regions for both experimental and filler sentences. As shown in the figure, AVC-SPR consistently produced the shortest reading times at early regions, and this trend continued through the critical and spillover regions (i.e., regions 11 or 12). However, reading times in AVC-SPR increased substantially near the end of the sentence. These patterns are consistent with rapid unmasking.

In the Introduction, I hypothesized that AVC-SPR may be particularly susceptible to rapid unmasking because readers can see how many key presses are required to reveal the entire sentence. The present results are consistent with this hypothesis.

In contrast to AVC-SPR, the other cumulative SPR tasks—NAVC-SPR and PC-SPR—showed clear garden-path and number-mismatch effects that were at least as well localized as those in NC-SPR. These findings suggest that omitting visual markers indicating upcoming word positions (NAVC-SPR/PC-SPR) and limiting the number of words that accumulate on the screen (PC-SPR) discourage rapid unmasking and encourage participants to attend to each word, at least as much as in NC-SPR.

It is important to note that, as shown in Fig. 8, reading times for both experimental and filler sentences are substantially shorter at earlier regions in NAVC-SPR (and PC-SPR) than in NC-SPR—a pattern also observed for AVC-SPR relative to NC-SPR. However, the data show that reading times become longer in NAVC-SPR than in NC-SPR once the critical regions are encountered. Although reading times in NAVC-SPR also become substantially longer towards the end of the sentence, resembling the pattern seen in AVC-SPR, the overall results suggest that this late increase does not reflect rapid unmasking. Instead, it more likely indicates that participants engage in rereading (e.g., in preparation for the comprehension question) as they approach the end of the sentence.

Another crucial finding is that the present study showed larger garden-path effects in NAVC-SPR (132 ms) and PC-SPR (94 ms) than in NC-SPR (41 ms), and larger number-mismatch effects in NAVC-SPR (84 ms) than in NC-SPR (29 ms). These findings point to a potential advantage of cumulative over non-cumulative presentation: when participants encounter processing difficulty, they may at least sometimes reread the sentence, and cumulative presentation can reflect this behavior. The results thus suggest that NAVC-SPR and PC-SPR may be more useful than NC-SPR for estimating the magnitude of processing difficulty when rereading plays a role (Rayner et al., 2004).

The study also showed that the estimated power to detect garden-path and number-mismatch effects was generally higher for NAVC-SPR than for NC-SPR. The high statistical power of NAVC-SPR is likely due, at least in part, to the large effect sizes observed in this task. This finding suggests that NAVC-SPR may be more effective than NC-SPR at detecting garden-path and number-mismatch effects.

Regarding comprehension accuracy for unambiguous and ambiguous sentences, the results showed lower accuracy for ambiguous sentences in all tasks. This finding replicates previous research on lingering misinterpretation (e.g., Slattery et al., 2013), suggesting that cumulative presentation can capture this phenomenon. In addition, comprehension accuracy was higher in all cumulative SPR tasks than in NC-SPR, suggesting another potential advantage of cumulative presentation: allowing rereading may improve sentence comprehension.

In summary, in terms of the four criteria—(i) effect detection, (ii) effect size, (iii) effect localization, and (iv) prospective statistical power—all tasks (NAVC-SPR, AVC-SPR, PC-SPR, and NC-SPR) were sensitive to both garden-path and number-mismatch effects. However, these effects were less robust or less localized in AVC-SPR. NAVC-SPR showed the largest effect sizes, followed by PC-SPR and NC-SPR. Power analysis further indicated that NAVC-SPR generally had the highest estimated power. Together, these findings suggest that NAVC-SPR may be the most powerful of the tasks tested for detecting garden-path and number-mismatch effects, and that cumulative SPR can be a viable alternative to non-cumulative SPR in sentence-processing research, contrary to the widely held view.

#### Revisiting Just et al. (1982)

The findings of the present study challenge the widely held view in sentence-processing research that cumulative presentation is ineffective for investigating real-time sentence processing. This view originates from Just et al. (1982), who compared cumulative and non-cumulative SPR times with eye-tracking data and found weaker correspondence for cumulative SPR times. However, their findings are not necessarily incompatible with the present study for two reasons.

First, Just et al. may have used a format similar to AVC-SPR. The present study suggests that AVC-SPR is the least reliable of the four tasks for investigating real-time sentence processing. If Just et al. used AVC-SPR, this could explain why they failed to observe certain effects, such as the influence of word frequency.

Second, the weaker correspondence between eye-tracking data and cumulative SPR times in Just et al.’s study may be due to their focus on word-level effects across all words in texts. As shown in Fig. 8, reading times in NAVC-SPR and AVC-SPR vary substantially with word position: they are relatively short at the beginning of a sentence but become much longer near the end. Accordingly, word-level effects may differ substantially depending on where the words appear. For example, if the word “*girl*” appears both at the beginning and at the end of a text, its reading times may differ substantially between the two positions, although factors such as word length and frequency are perfectly controlled.

Importantly, the approach taken by Just et al. (1982)—examining word-level effects across all words in texts—is atypical in sentence-processing research. Sentence-processing studies generally control for positional effects, as in the present study, to ensure that reading-time differences are not confounded by word location within a sentence or text. Therefore, in well-controlled sentence-processing experiments, NAVC-SPR (and PC-SPR) may be a viable alternative to NC-SPR, whereas NC-SPR may be more suitable for research that investigates word-level effects across all words in a sentence or text.

## Conclusion

The present study examined the utility of three cumulative SPR variants relative to non-cumulative SPR. The aim was to test the widely held belief in sentence-processing research that cumulative presentation is ineffective for investigating real-time sentence processing. The results showed that cumulative presentation may be less reliable than non-cumulative presentation when upcoming word positions are visually indicated. However, cumulative presentation was as reliable as or more reliable than non-cumulative presentation when upcoming word positions were not indicated or when the number of words allowed to remain visible on the screen was limited. These findings challenge the widely held view that cumulative presentation is unsuitable for investigating real-time sentence processing and suggest that some cumulative formats are, at the very least, viable alternatives to non-cumulative SPR.

## Data Availability

Data, analysis code, and experimental materials are available at https://osf.io/p2rck and https://github.com/HirokiFujita1126/Cumulative-NonCumulativeSPRTask.

## Footnotes

1 In sentence-processing research, it is common to remove extremely fast or slow reading times because such values may be physically implausible (readers need some time to recognise a word and integrate it with the preceding context) or unlikely to reflect the targeted cognitive processes (under typical experimental conditions, it is unusual for a word to take an exceptionally long time to process). There is no consensus on how extreme values should be defined, so the criteria vary across studies. In SPR experiments, lower bounds are often around 200 ms, partly because fixations during normal reading typically last about 200–250 ms (Rayner & Pollatsek, 1989). Upper bounds are more variable, often ranging from 2000 to 10,000 ms (e.g., Aoshima et al., 2004; Arnett & Wagers, 2017; Cunnings & Fujita, 2021; Fujita, 2025c; Fujita & Cunnings, 2023; Hopp, 2006; Kobzeva & Kush, 2024; Kush et al., 2026; Kush & Dillon, 2021; Paolazzi et al., 2019; Parker et al., 2015; Roberts & Felser, 2011; Sturt et al., 1999; Sturt & Kwon, 2024; Wagers & Phillips, 2009). Researchers may be relatively permissive with upper bounds because reading times are commonly log-transformed, which reduces the influence of extremely slow values. Some studies also—or instead—exclude observations more than two to three standard deviations from the mean, although this approach can be problematic (see Harald Baayen & Milin, 2010; Nicklin & Plonsky, 2020; Ratcliff, 1993).
